# Effects of Carbon Nanoparticles and Chromium Combined Exposure in Native (*Ruditapes decussatus*) and Invasive (*Ruditapes philippinarum*) Clams

**DOI:** 10.3390/nano13040690

**Published:** 2023-02-10

**Authors:** Julieta Sturla Lompré, Lucia De Marchi, João Pinto, Amadeu M. V. M. Soares, Carlo Pretti, Federica Chielini, Eduarda Pereira, Rosa Freitas

**Affiliations:** 1 Center for the Study of Marine Systems (CESIMAR-CONICET), National Patagonian Center, Bv. Almte Brown 2915, Puerto Madryn 9120, Argentina; 2 Department of Biology & CESAM, University of Aveiro, 3810-193 Aveiro, Portugal; 3 Department of Chemistry and LAQV-REQUIMTE, University of Aveiro, 3810-193 Aveiro, Portugal; 4 Interuniversity Consortium of Marine Biology of Leghorn “G. Bacci”, 57128 Livorno, Italy; 5 Department of Chemistry and Industrial Chemistry, University of Pisa, Udr INSTM Pisa, 56126 Pisa, Italy

**Keywords:** bivalves, chromium, carbon nanotubes, bioaccumulation, oxidative stress, cellular biomarkers, multiple contaminants

## Abstract

Studies have described the occurrence of nanoparticles (NPs) in aquatic ecosystems, with particular attention to the widely commercialized carbon nanotubes (CNTs). Their presence in the environment raises concerns, especially regarding their toxicity when co-occurring with other pollutants such as metals. In the present study, changes to the metabolic capacity, oxidative, and neurologic status were evaluated in the presence of carboxylated multi-walled CNTs and chromium (Cr(III)) using two of the most ecologically and economically relevant filter feeder organisms: the clam species *Ruditapes decussatus* and *R. philippinarum*. Results indicated that although Cr, either alone or in combination with CNTs, was found in a similar concentration level in both species, a species-specific Cr accumulation was observed, with higher values in *R. decussatus* in comparison with *R. philippinarum*. Inhibition of antioxidant defenses and neurotoxic effects were detected only in *R. philippinarum*. The interaction between contaminants seems to have no effect in terms of antioxidant enzyme activities and neuro status. Nevertheless, synergistic activation of responses to both contaminants may have altered the metabolic capacity of bivalves, particularly evident in *R. decussatus*. While both clams are tolerant to both contaminants (alone and together), they showed a relevant accumulation capacity, which may represent a possible contaminant transfer to humans.

## 1. Introduction

Chromium (Cr) is a very stable metal, presenting different forms in nature, such as divalent (Cr(II)), trivalent (Cr(III)), and hexavalent (Cr(VI)) forms [1]. This metal occurs naturally in water either in the Cr(III) or Cr(VI) oxidation state, with Cr(VI) being known as a highly toxic form of this metal with severe impacts on the environment [2], thus representing a risk for coastal ecosystems [3]. Due to its easy permeability through the cell membrane, several Cr(VI)-toxicological effects have been documented in marine invertebrate communities, including decreased survival [4], enhancement of antioxidant and biotransformation defenses [5], changes in the transcription of different genes [6], impairment of functional and molecular immune parameters [7], lysosomal membranes destabilization [8], changes on lipid and carbohydrate metabolism and the expression of estrogen-responsive genes [8], DNA injury and suppression of protein synthesis [9]. In contrast, Cr(III) is less membrane-permeable and is considered an essential trace metal, being involved in metabolic processes and nucleic acid synthesis. However, it has also been identified as a potential hazard in aquatic environments, when available in high concentrations [10]. There is evidence that, when able to pass through the cell membrane, Cr(III) can induce damage to cell organelles [11]. As such, and with the increasing concern regarding Cr contamination, more studies are required to assess the impact of Cr(III) on aquatic environments. While the World Health Organization (WHO) estimated that the Cr concentration in surface water can vary between 1 and 10 μg/L [12], this value can be significantly affected by the discharge of contaminated effluents. Coastal Cr pollution is mainly due to anthropogenic sources such as effluents from tanneries, textiles, mining, electroplating, dyeing, printing, photographic printing, pharmaceuticals, stainless steel manufacturing, and rubber manufacturing industries [13]. Cr-rich effluents usually contain a mixture of Cr(III) and Cr(VI) and, among these, Cr(III) from tannery effluents represents a major share of widespread contamination in water and soil [14]. Effluent regulations for Cr(III) vary from 1 to 5 mg/L (in the case of direct discharge into water bodies), however, values as high as 1500 to 3000 mg/L can be found on tannery effluents [15]. These effluents may then contribute to the contamination of aquatic systems, which represents a risk to both human and environmental health. For example, aquaculture waters in the vicinity of large cities may be at risk of contamination from surrounding industrial and agricultural activity. Ngoc et al. [16] reported Cr values from 0.32 to 4.32 mg/L in aquaculture waters in Thủy Nguyên in Northern Vietnam, which is close to a shipbuilding company, thermal power, and cement plant as well as a stone mine. In Lake Taihu, one of the most industrialized areas in China considered an important source for fisheries, Cr was found up to 3.94 µg/L in aquaculture waters [17].

In addition to the increasing presence of trace elements in the environment, several studies have demonstrated the growing occurrence of contamination by nanomaterials. Nanomaterials have been considered crucial in different areas, such as nanomedicine, technology, the textile industry, computer science, energy sector, among others. The exponential increase in their use has led to the need for studies that can identify their possible adverse effects on organisms [18]. Due to specific properties such as exceptional electrical and thermal conductivity and higher mechanical strength than traditional materials, carbon nanotubes (CNTs) are considered to be one of the most important and commercialized nanomaterials [19]. So far, worldwide CNTs production values have been estimated to range from 350 tons/year to 500 tons/year [20] and their release has increased substantially over the last decades. Interestingly, Petersen et al. [21] showed release pathways of CNTs into the natural environment, with particular concern for the aquatic ones, which tend to be the ultimate sink for this type of contaminant [22]. The authors reported that frequent sources are related to incineration, disposal in landfills, or wastewater as a result of poor removal efficiency in wastewater treatment plants. It is now recognized that the potential biological implications of CNTs are related to their behavior in solution, which displays a tendency to aggregate and form clusters that exhibit colloidal behavior [23]. In particular, although individual CNT molecules are insoluble, formed aggregates are stable under certain environmental conditions, which in turn can intensify the risk of exposure and toxicity [24]. While the usefulness of these materials is undeniable, their design and application should be sustainable and, therefore, environmentally benign. As such, evaluating the impacts of these materials and their interactions with other anthropogenic contaminants is fundamental for their environmentally safe application. Information regarding CNT’s toxic effects is available on different invertebrate species, such as bivalves, freshwater snails, brine shrimp, polychaetes, and microcrustaceans [25,26,27,28,29,30]. The effects of these nanomaterials include metabolic alterations, oxidative stress induction, cellular damage, neurotoxicity, reproductive damage, genotoxicity, and DNA injury [27,31,32,33,34].

Despite the concerns regarding both trace metals and nanomaterials contamination, no studies have yet reported the potential synergistic effects of both classes of contaminants in aquatic fauna. After release, NPs can interact with other chemicals already present in the environment, leading to a co-exposure of organisms and the occurrence of combined effects [35,36], defined as Trojan-horse effects. For CNTs in particular, these NPs could adsorb metals, acting as carriers and, in turn, lead to a facilitated uptake of chemicals into organisms, resulting in a toxicity increase [35]. Combined effects of CNTs and individual metals such as Cr could lead either to antagonistic effects (two compounds compete for the same binding site) or to synergistic effects (one compound promotes the uptake of another) [36]; however, these interactions remain to be studied. The present study aims to address this gap in the literature by examining the effects of co-exposure to Cr and CNT (two of the most common contaminants in their classes) in two bivalve species: *Ruditapes decussatus* and *Ruditapes philippinarum*. The choice of the studied species is based on the fact that, in Portugal, these are the most successfully commercialized edible clams, representing a major pathway to the bioamplification of contaminants along the food chain. The native *R. decussatus* spatial distribution includes European and Mediterranean coastal waters [37], while the invasive species *R. philippinarum* was introduced for culture in the same area [38,39]. Due to their similar morphologies, feeding and habitat preferences, the introduction of the invasive clam led to the competition between both species in natural conditions [40], including in the Ria de Aveiro, Portugal [41]. Previous studies have already demonstrated that these closely related species may respond differently to the same environmental stressor, including to pollutants [42,43,44]. The adverse effects of both contaminants, acting individually or in combination (Cr, CNTs, CNTs + Cr) in the clams, were analyzed by comparing biochemical responses in both species. Specifically, we evaluate if: i) the exposure to Cr and CNTs affects the physiological and oxidative profiles of both clams, ii) the toxic impacts caused by the co-exposure of pollutants could generate interactive effects, and iii) the possible toxic impacts can be species-specific. To meet these hypotheses, a set of biomarkers was developed to evaluate the metabolic capacity, oxidative stress, and neurotoxic status of clams after a chronic exposure period.

## 2. Materials and Methods

### 2.1. Chemical Description

A chromium (Cr) concentration of 3.5 mg/L was selected based on concentrations found in highly contaminated systems (with regulations broadly limiting the discharge limit to 1–5 mg/L), to simulate a scenario of anthropogenic contamination [15]. For this experiment, Cr(III) was used. This form of Cr is generally considered less toxic than its Cr(VI) counterpart but still presents a wide distribution in the aquatic environment (see references above). For this reason, Cr(III) was considered more appropriate for this study, in particular for the evaluation of the synergistic effects of Cr + CNTs. Functionalized multi-walled carbon nanotubes (MWCNTs) were used in the present study by adding carboxyl groups (-COOH) to the CNTs (TNMC1 series, http://www.timesnano.com, (accessed on 1 January 2023)). These groups ionize in water charging the oxygen atoms negatively in the aqueous phase and the electrostatic repulsive forces between negative surface charges of the oxygen-containing groups can lead to the stability of oxidized CNTs in the seawater column [45]. The large specific surface area may facilitate pollutant adhesion and thus influence CNT toxicity in itself and/or toxicity of co-pollutants and influence the bioaccumulation of environmental contaminants [46]. The concentration of 200 μg/L was chosen considering environmentally relevant concentrations, predicted values in the environment, and previous studies that assessed the effects caused in aquatic species [30,47,48].

### 2.2. Experimental Conditions

*R. decussatus* and *R. philippinarum* specimens were sampled from the Ria de Aveiro (northwest coast of Portugal) and immediately transported to the laboratory. Before the experiment, clams were acclimated for 10 days in artificial seawater (salinity 30) under continuous aeration, natural photoperiod, and constant temperature (17 ± 1 °C) and pH (8.0–8.2). After the first three days in the laboratory and during the experimental time, clams were fed with Alga Mac Protein Plus (15 × 10^4^ cells *per* animal *per* day—Aquafauna Bio-Marine) every 2–3 days.

After acclimation, organisms were subjected to different treatments for 28 days: control (CTL), where no exposure was tested; carbon nanotubes exposure (CNTs); chromium exposure (Cr); and the combined exposure of both CNTs and Cr (CNTs + Cr). Each treatment was represented by three aquaria, each with 4 organisms (12 organisms *per* treatment). Stock solutions of 50 mg/L CNTs were prepared as described by Sturla Lompré et al. [49]. Chromium spiking was performed using an intermediate solution obtained from the dilution of a stock solution of 1000 mg/L (Cr(III), Inorganic VenturesTM). The stability of Cr(III) in seawater had already been evaluated by Henriques et al. [50] for a solution of 2 mg/L obtained from an identical stock solution. Water was renewed every week to re-establish CNTs and Cr concentrations and physical parameters were checked every day. Water samples were collected from each aquarium every week after spiking to determine the real Cr exposure concentration and before water renewal for CNTs characterization. After exposure, clams were frozen with liquid nitrogen for Cr quantification as well as biochemical parameters determination. During the exposure time, no mortality was recorded.

### 2.3. Carbon Nanotubes Characterization

To verify the aggregation state of CNTs, three analyses *per* sample were performed by dynamic light scattering (DLS) using a DelsaTM NanoC Particle Size Analyzer (Beckman Coulter) (detection angle = 166.22°). Each analysis was carried out by performing 120 acquisitions. Briefly, water samples (50 mL *per* replicate) were taken from each aquarium at different exposure periods: T7—water samples collected after one week of exposure; T14—water samples collected after two weeks of exposure; T21—water samples collected after three weeks of exposure; T28—samples collected after four weeks of exposure. Particle size distribution and distribution averages were carried out using CONTIN analysis routines by Delsa Nano 3.73 software. The mean size of the suspended particle aggregates and polydispersity index (PDI) of the dispersions were calculated by the cumulate method.

### 2.4. Chromium Determination in Seawater and Organisms

Water samples were diluted by a factor of 10 in a matrix of HNO_3_ 1% before analysis. Chromium quantification was performed by Inductively Coupled Plasma Optic Emission Spectrophotometry (ICP-OES) in a HORIBA Jobin Yvon, Activa M spectrometer. Calibration curves were built with five standards ranging from 0.01 mg/L to 1 mg/L, prepared by successive dilutions of a commercially certified standard for ICP analysis (Inorganic Ventures^TM^). Calibration curves were rejected if correlation coefficients were lower than 0.999 or if the variation coefficient between standards exceeded 10% (n = 3). The lowest standard of 0.01 mg/L was considered the limit of quantification. For tissue quantification, the clam’s whole soft tissue (two individuals *per* aquarium, six *per* treatment) was homogenized and subjected to acid digestion using a microwave CEM MARS 5. Homogenized tissue samples (200 mg dry weight, DW) were added to previously washed Teflon tubes, along with 1 mL of H_2_O, 1 mL of HNO_3_ (65%), and 2 mL of H_2_O_2_ (30%), and subjected to high temperature and pressure conditions (15 min ramp to 170 °C, which was then held for 5 more min). The final sample was diluted to a final volume of 25 mL using ultrapure water (18 MΩ/cm). The lowest standard of 0.01 mg/L was considered the limit of quantification.

### 2.5. Biomarker Responses

After the 28-day experimental period, soft tissues of frozen clams (4 *per* aquarium) were homogenized using liquid nitrogen, divided into 0.2 g aliquots (fresh weight, FW), and used to investigate biochemical alterations. To this end, clams’ metabolic capacity, cellular damage, antioxidant and biotransformation defenses, and neurological status were determined. Metabolic capacity biomarkers included electron transport system activity (ETS) and protein (PROT) content. Cellular damage was evaluated by measuring lipid peroxidation (LPO) levels. Antioxidant and biotransformation defenses were investigated by measuring the activity of the enzymes superoxide dismutase (SOD), catalase (CAT), glutathione peroxidase (GPx), and glutathione S-transferase (GSTs). Finally, neurological status was evaluated by assessing acetylcholinesterase (AChE) activity. See Supplementary Material (S1) for detailed methodologies.

### 2.6. Statistical Analysis

Statistical differences in chromium concentrations and biochemical parameters in clam tissues were evaluated using PERMANOVA+ add-on in PRIMER v6 [51]. When the main test revealed statistically significant differences (*p* < 0.05), pairwise comparisons were performed. Data obtained from biochemical analyses and Cr concentrations were submitted to the following null hypotheses for each biomarker: (a) for each species, no significant differences exist among treatments; and (b) for each treatment, no significant differences exist between species.

## 3. Results

### 3.1. Carbon Nanotubes Characterization

Performed DLS analysis showed a discontinuous trend of CNTs aggregation state along different exposure periods as well as between exposure treatments. In detail, after 7 days, the presence of suspended materials in aqueous media was not detected in both treatments (CNTs and CNTs + Cr) and species (*R. decussatus* and *R. philippinarum*) (data not shown). By contrast, in *R. decussatus*, after 14 days, the largest NP aggregates were detected in CNTs + Cr treatment, while an opposite behavior was observed after 21 and 28 days, showing the highest particle aggregation state under CNTs treatment alone (Table 1). Considering *R. philippinarum*, CNTs treatment alone showed larger particle aggregates compared to the combined one after 14 days. Instead, after 21 and 28 days, the largest NP size was identified under CNTs + Cr treatment. These results confirmed that suspended materials showed an opposite behavior not only between species but also between treatments during the exposure period.

### 3.2. Chromium in Seawater and Organisms

Chromium levels in seawater immediately after the spiking were 3.21 ± 0.37 mg/L and 3.15 ± 0.53 mg/L for the aquaria containing *R. decussatus* and *R. philippinarum*, respectively. In CTL treatments, Cr levels were lower than the quantification limit of 0.01 mg/L, proving the absence of contamination in the control aquaria (Table 2).

Chromium was also quantified in clams’ tissues at the end of 28-day exposure (Table 3). In both species, Cr concentrations were higher in specimens exposed to this element in comparison to those exposed to CTL and CNTs alone, but no significant differences were observed between Cr and CNTs + Cr treatments (Table 3). Although in the CNTs + Cr mixture, *R. philippinarum* tissues tended to accumulate higher Cr concentrations than in Cr exposed alone, these differences were not significant. In both treatments (CNTs + Cr and Cr), *R. decussatus* accumulated significantly more Cr than *R. philippinarum*.

### 3.3. Biological Responses

#### 3.3.1. Metabolic Activity and Energy Reserves

**Figure 1:**
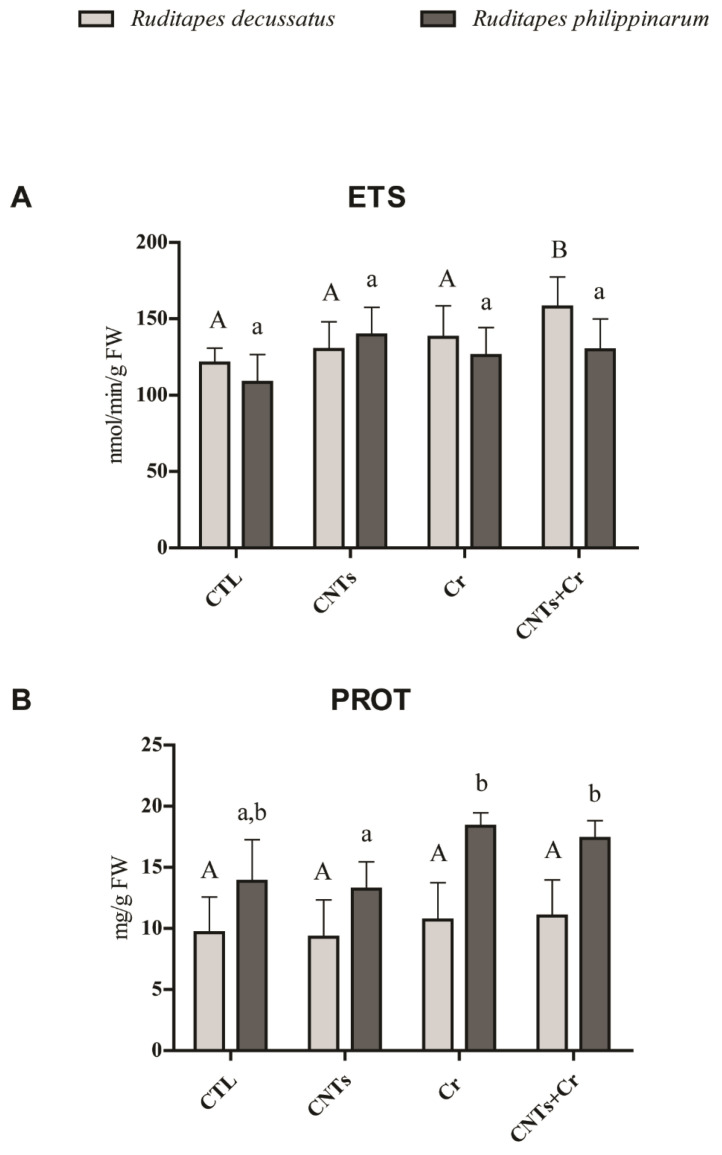
(**A**) Electron transport system (ETS) activity; (**B**) protein (PROT) content (mean values + standard deviation), in *Ruditapes decussatus* (light gray bars) and *Ruditapes philippinarum* (dark gray bars) after a 28-day exposure period. For each species, tested treatments were: control (CTL), carbon nanotubes (CNTs), chromium (Cr), and the combination of both contaminants (CNTs + Cr). Significant differences (*p* < 0.05) among treatments are represented with different letters (lowercase for *R. decussatus* and uppercase for *R. philippinarum*).

Significantly higher metabolic activity was only observed in *R. decussatus* exposed to CNTs + Cr compared to the remaining treatments, while ETS activity in *R. philippinarum* was not affected by any of the treatments. No significant differences between species were observed (Figure 1A). Exposed *R. decussatus* specimens presented no significant differences in PROT concentration in relation to non-exposed organisms. Similarly, exposed *R. philippinarum* did not differ from the control either, but in the presence of Cr (acting alone or combined), higher PROT content was observed. Between species, there were no significant differences (Figure 1B).

#### 3.3.2. Indicators of Cellular Damage

**Figure 2:**
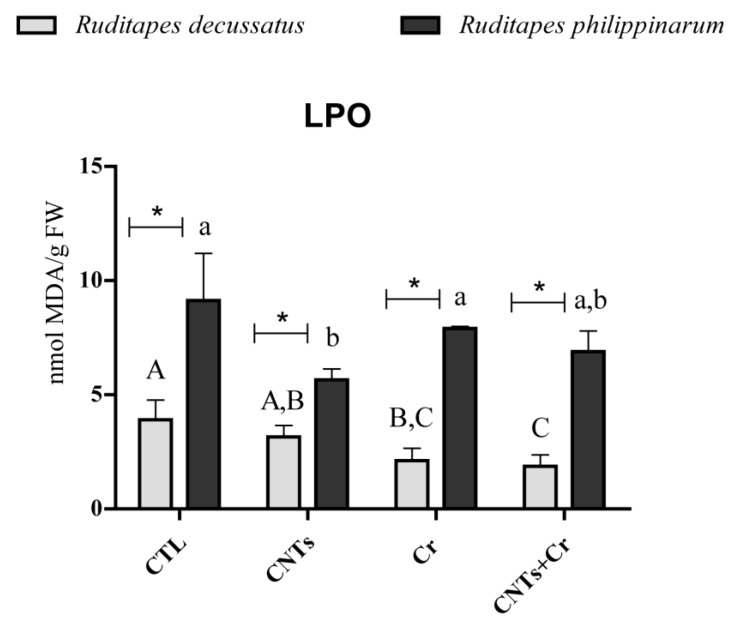
Lipid peroxidation (LPO) levels (mean values + standard deviation), in *Ruditapes decussatus* (light gray bars) and *Ruditapes philippinarum* (dark gray bars) after a 28-day exposure period. For each species, tested treatments were: control (CTL), carbon nanotubes (CNTs), chromium (Cr), and the combination of both contaminants (CNTs + Cr). Significant differences (*p* < 0.05) among treatments are represented with different letters (lowercase for *R. decussatus* and uppercase for *R. philippinarum*). For each treatment, significant differences (*p* < 0.05) between *R. decussatus* and *R. philippinarum* are represented with an asterisk (*).

*R. decussatus* exposed to Cr (Cr and CNTs + Cr treatments) revealed a significant decrease in LPO levels compared to CTL. No significant cellular damage was identified either in *R. philippinarum* contaminated with Cr, individually or in combination (Cr and CNTs + Cr). Comparing clam species, *R. philippinarum* presented significantly higher LPO levels compared to *R. decussatus* in all groups (Figure 2).

#### 3.3.3. Antioxidant and Biotransformation Defenses

**Figure 3:**
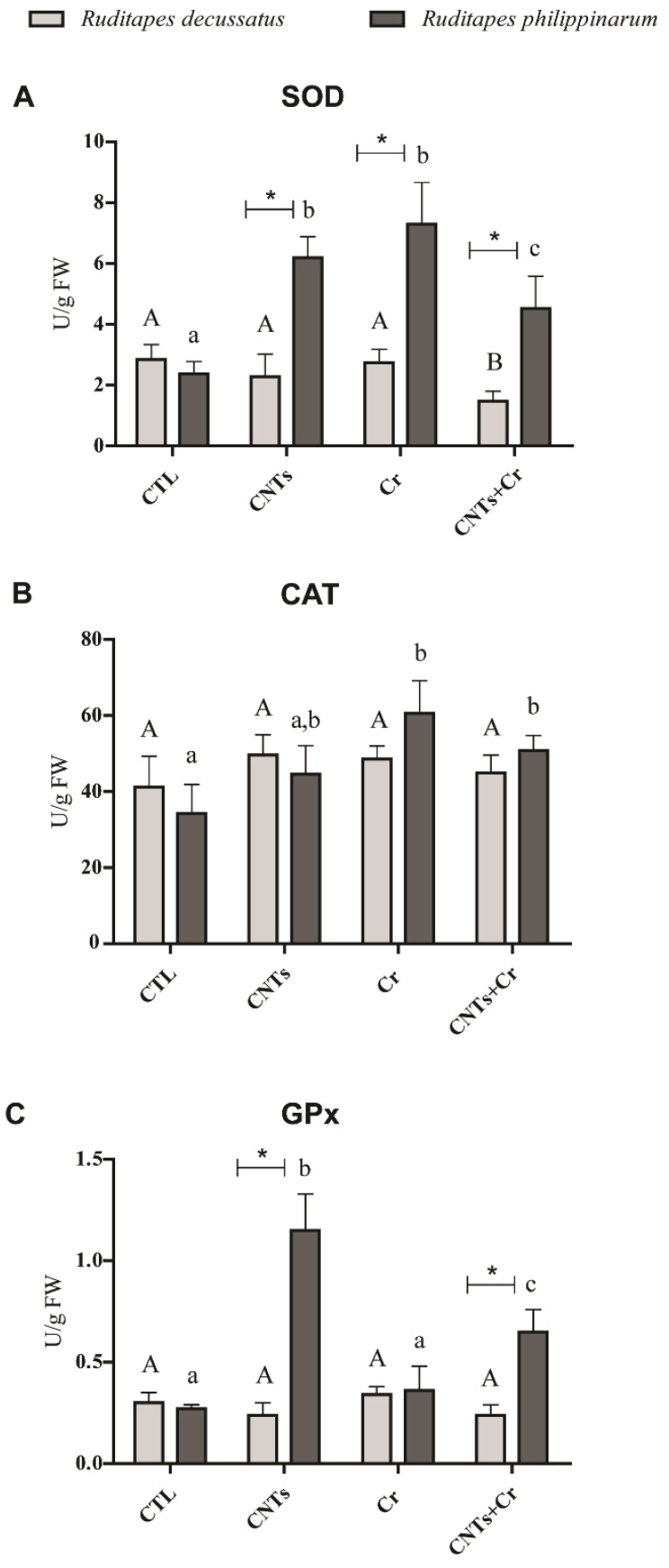
(**A**) Superoxide dismutase (SOD); (**B**) Catalase (CAT); **(C)** Glutathione peroxidase (GPx) activities (mean values+ standard deviation), in *Ruditapes decussatus* (light gray bars) and *Ruditapes philippinarum* (dark gray bars) after a 28-day exposure period. For each species, tested treatments were: control (CTL), carbon nanotubes (CNTs), chromium (Cr), and the combination of both contaminants (CNTs + Cr). Significant differences (*p* < 0.05) among treatments are represented with different letters (lowercase for *R. decussatus* and uppercase for *R. philippinarum*). For each treatment, significant differences (*p* < 0.05) between *R. decussatus* and *R. philippinarum* are represented with an asterisk (*).

In *R. decussatus* exposed to CNTs + Cr, the activity of SOD was significantly lower compared to the remaining treatments (Figure 3A). In *R. philippinarum* the activity of this enzyme showed a significant increase in contaminated clams compared to CTL ones. Comparing species, *R. philippinarum* presented significantly higher SOD activity than *R. decussatus* regardless of the treatment (Figure 3A). Considering *R. decussatus* no variation in CAT activity was detected among treatments, whereas in *R. philippinarum,* all treatments generated activation of this enzyme, with significant differences between non-contaminated clams and the ones exposed to Cr and CNTs + Cr. No differences in CAT activity were registered between the two species in any of the treatments (Figure 3B).

**Figure 4:**
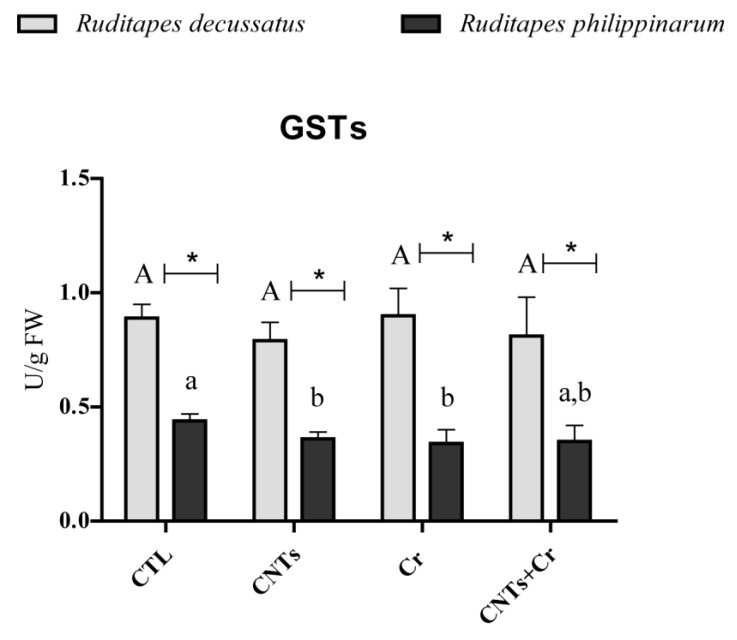
Glutathione S-transferases (GSTs) activity (mean values+ standard deviation), in *Ruditapes decussatus* (light gray bars) and *Ruditapes philippinarum* (dark gray bars) after a 28-day exposure period. For each species, tested treatments were: control (CTL), carbon nanotubes (CNTs), chromium (Cr), and the combination of both contaminants (CNTs + Cr). Significant differences (*p* < 0.05) among treatments are represented with different letters (lowercase for *R. decussatus* and uppercase for *R. philippinarum*). For each treatment, significant differences (*p* < 0.05) between *R. decussatus* and *R. philippinarum* are represented with an asterisk (*).

Similarly, GPx activity in *R. decussatus* did not differ significantly among all treatments, while it significantly increased in *R. philippinarum* exposed to CNTs alone and CNTs + Cr. Comparing species, *R. philippinarum* presented significantly higher enzyme activity in the presence of CNTs and CNTs + Cr than *R. decussatus* (Figure 3C). Similar GSTs activity in *R. decussatus* was found between CTL and exposure treatments. By contrast, in *R. philippinarum*, significantly lower enzyme activity was registered in CNTs and Cr treatments compared to the control. Comparing species, *R. decussatus* showed significantly higher GSTs activity regardless of the treatment (Figure 4).

#### 3.3.4. Neurotoxicity

**Figure 5:**
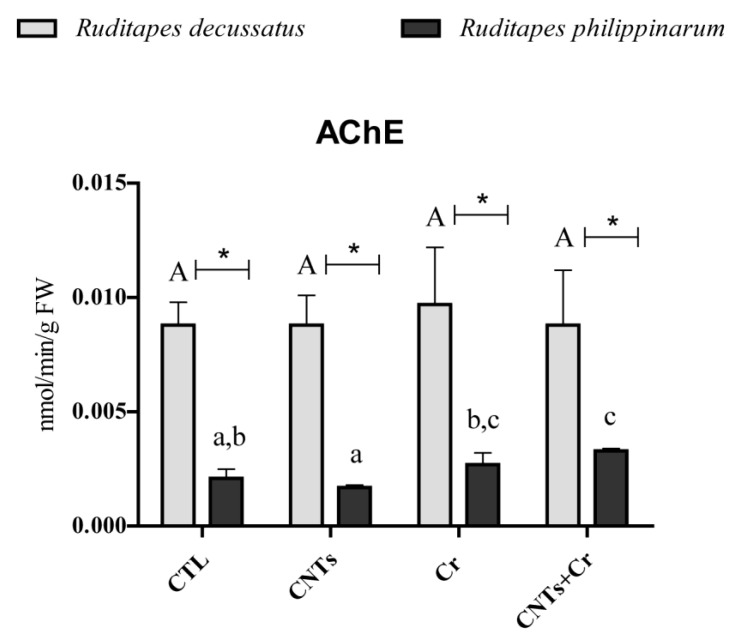
Acetylcholinesterase (AChE) activity (mean values+ standard deviation), in *Ruditapes decussatus* (light gray bars) and *Ruditapes philippinarum* (dark gray bars) after a 28-day exposure period. For each species, tested treatments were: control (CTL), carbon nanotubes (CNTs), chromium (Cr), and the combination of both contaminants (CNTs + Cr). Significant differences (*p* < 0.05) among treatments are represented with different letters (lowercase for *R. decussatus* and uppercase for *R. philippinarum*). For each treatment, significant differences (*p* < 0.05) between *R. decussatus* and *R. philippinarum* are represented with an asterisk (*).

The activity of AChE in *R. decussatus* showed no significant differences among test groups, while *R. philippinarum* exhibited significantly higher activity when clams were exposed to Cr in combination with CNTs. Comparing species, *R. decussatus* presented higher values than *R. philippinarum* at all treatments (Figure 5).

## 4. Discussion

The present findings revealed a similar accumulation of Cr either alone or in combination with CNTs, especially noticed in *R. decussatus*, indicating that CNTs may not act as a carrier of Cr. Although the literature has shown that CNTs may facilitate the transport of other elements [35], different authors, analyzing the interaction between CNTs and other compounds, observed that the bioconcentration of the two chemicals was independent [34,49,52]. A review by Anastopoulos et al. [53] reports that Cr(III) can be adsorbed onto the surface of CNTs to some extent. The reported sorption efficiency of this material is higher towards Cr(VI) at lower pH (2–4). In contrast, the removal of Cr(III) has shown to be more substantial at higher pH (5–8), which raises the possibility of Cr(III) adsorption onto CNTs under the experimental conditions of the present study. However, previous studies have only evaluated this sorption capacity using simplistic matrices such as deionized water (See Atieh et al. [54]). Seawater is an exceptionally complex medium that can be a detriment to the efficiency of many adsorption processes due to the increase in ionic strength and competition with other elements onto the binding sites of the material [55,56]. Since the sorption ability of Cr(III) by the CNTs was not evaluated under the exact experimental conditions, it is not possible to correctly predict the degree to which these interactions may occur; however, this possibility cannot be excluded. On the other hand, different Cr content between species was observed, with higher metal accumulation in *R. decussatus* compared to *R. philippinarum*. Bivalves stay isolated from the environment by maintaining their valves closed, which reduces their filtration and thus prevents accumulation of pollutants in their tissues [57]. A decrease in filtration capacity is related to an energy-saving mechanism, associated with shell closure. Although both species did not differ in metabolic capacity (measured through electron transport system (ETS) activity), *R. decussatus* showed slight increases than *R. philippinarum* in the presence of Cr, especially when combined with CNTs, which may have contributed to higher Cr concentration in the native species. Furthermore, even though in the present study, *R. decussatus* was the species with the highest detoxification capacity, this capacity was similar regardless of the presence or absence of Cr, as showed by *R. philippinarum,* which might indicate the limited role of GSTs on Cr and CNTs detoxification.

Despite the similar accumulation of Cr when alone or in combination with CNTs, the interaction between these two contaminants may synergistically activate signaling pathways in response to adverse conditions, which may explain higher metabolism in *R. decussatus* clams exposed to CNTs + Cr. Higher metabolic activity in response to a combination of xenobiotics was also documented by Britto et al., 2020 [58]. The authors observed an increase in ETS activity when *R. philippinarum* was contaminated with copper (Cu) together with graphene oxide (GO) under a 7.3 pH level, fueling up filter-feeder defense mechanisms. The results here presented also demonstrated that the PROT content was maintained (*R. decussatus*) or even increased (*R. philippinarum*) in the presence of contaminants which might indicate that clams increased the production of proteins, namely enzymes, to enhance their defense mechanisms (e.g., increase in the number of antioxidant enzymes) and/or might indicate that clams were capable of preserving this energy source using others such as glycogen or lipids. This behavior was already reported by Sokolova et al. [59] as a predominant adaptive strategy of metabolic responses (energy conservation) that allows an invertebrate organism to survive environmental disturbances.

Results of metabolic alterations were accompanied by variations in enzyme activities, namely antioxidant and detoxification ones, to fight against the stress induced by CNTs and Cr (alone or combined) and to avoid damage caused by ROS generation. Briefly, ROS production is a physiological process that is maintained in normal function by superoxide-dismutase (SOD) [60]. This enzyme is directly responsible for the removal of the superoxide anion (O_2_^-^) with the formation of hydrogen peroxide (H_2_O_2_) that can be used by catalase (CAT) or glutathione peroxidases (GPx) enzymes [61]. Under stressful conditions, ROS can be overproduced, and in response, bivalves are known to activate antioxidant defenses (e.g., [47,52,62,63,64,65,66,67]). A similar response was described here for *R. philippinarum* exposed to both contaminants showing increased antioxidant activity compared to control clams in an attempt to eliminate ROS and mostly to prevent cellular damage (e.g., LPO). In fact, under these conditions, no LPO was observed. Similarly, Mesquita et al. [68] observed the enhancement of antioxidant defenses in the bivalves *Cerastoderma edule* and *Scrobicularia plana* when exposed to Cu. However, in this case, the antioxidant process was not effective, with the occurrence of increased LPO levels. The results obtained in the present study confirmed the efficiency of the antioxidant enzymes in the fight against the LPO, which may explain a higher tolerance of *R. philippinarum* to contaminants, as reported by other researchers. This hypothesis was also confirmed by results obtained in our previous study [49]. Using the same species, the authors exposed *R. philippinarum* clams to CNTs, Terbium (Tb), and the combination of both contaminants and observed no cellular damage as a consequence of a greater antioxidant activation system. An opposite antioxidant behavior was observed in *R. decussatus*. Indeed, CAT and GPx activities resulted unaltered in comparison with their respective controls, while SOD activity showed a decreasing trend when clams were exposed only to CNTs + Cr. The discrepancy in biochemical responses between the two species may be explained by the different species’ sensitivity to contaminants; while in contaminated *R. philippinarum* the antioxidant mechanisms were activated, preventing LPO levels, in *R. decussatus* neither the enzymatic defense was stimulated, nor cellular damage was observed. Considering that similar biochemical responses were shown previously in both species exposed to Tb and CNTs acting alone and together [49], the present results corroborated the higher tolerance of the native species *R. decussatus* to different types of contaminants compared to the invasive clam (*R. philippinarum*). When contaminated, bivalves may also increase the activity of glutathione S-transferase (GSTs), a group of enzymes involved in detoxification [69,70,71]. However, in the present study, clams exposed to both contaminants showed a slight decrease (*R. philippinarum*) and unaltered (*R. decussatus*) activity of GSTs, which could indicate that this group of enzymes was not involved in the biotransformation of the tested contaminants into less toxic substances.

Acetylcholinesterase (AChE) is an important enzyme in the neural system, catalyzing the hydrolysis of the neurotransmitter acetylcholine [72]. The inhibition of this enzyme has been recognized as a biological marker of different contaminants such as metals and NPs [47,52,67,73,74,75,76,77]. However, there is also evidence of its increases in marine organisms, such as in *Perna viridis* in the presence of arsenic, lead, and cadmium, and in *R. decussatus* and *R. philippinarum* in the presence of terbium combined with CNTs [49,78,79,80]. The results obtained are in agreement with Liu et al. [80], who reported an increase in AChE in *Ruditapes philippinarum* exposed to Hg. Bainy et al. [78] suggested that the increase could be due to a new enzyme synthesis after being initially inhibited, and Romani et al. [81] proposed that there might be an enhancement of the formation of the enzyme–substrate complex increasing the activity of AChE. The effects of the increase in the synthesis of AChE following CNTs exposure combined with the rare earth element Tb were also described by Sturla Lompré et al. [49] not only in *R. philippinarum* but also in *R. decussatus*. On the contrary, in the present study, only in *R. philippinarum*, which resulted in the most sensitive species, AChE activity increased when exposed to both CNTs and Cr, probably due to the interaction of both contaminants with the AChE-substrate complex. In the case of *R. decussatus*, no changes in AChE were observed, reinforcing the tolerance of this species to the tested pollutants.

## 5. Conclusions

This study demonstrates different Cr content between species, with higher metal accumulation in *R. decussatus* compared to *R. philippinarum*. However, biological responses were mostly detected in *R. philippinarum*, with *R. decussatus* being the most tolerant species. Moreover, the interaction between CNTs and Cr seems to have no effect in terms of antioxidant enzyme activities and neuro status. Nevertheless, synergistic effects may have changed the metabolic capacity of clams. Bivalves play key function and economic roles in the coastal environment, and assessing mechanisms behind specie’s responses to mixtures of pollutants appears paramount for sustaining marine biodiversity. Moreover, while both clams were tolerant to both contaminants at used concentrations, they showed a relevant accumulation capacity, which, in turn, may suggest a possible contaminant transfer to secondary consumers, including humans.

## Figures and Tables

**Table 1:** Dynamic light scattering (DLS) results of size (nm) and polydispersity index (PDI) of carbon nanotubes (CNTs) alone and in combination with chromium (CNTs+ Cr) suspensions collected along different exposure times (days 7 (T7), 14 (T14), 21 (T21), and 28 (T28)) and tests (*Ruditapes decussatus* and *Ruditapes philippinarum*). I.d.: not detected colloidal material in the analyzed sample at the end of 120 acquisitions).

	**T14**			
	**CNTs**		**CNTs + Cr**	
	**Size (nm)**	**PDI**	**Size (nm)**	**PDI**
*R. decussatus*	I.d.	-	I.d.	-
	1215.4	0.49	I.d.	-
	I.d.	-	7387.2	3.10
*R. philippinarum*	I.d.	-	I.d.	-
	I.d.	-	I.d.	-
	4201.9	1.88	2279.8	1.02
	**T21**			
	**CNTs**		**CNTs + Cr**	
	**Size (nm)**	**PDI**	**Size (nm)**	**PDI**
*R. decussatus*	9688.5	3.99	I.d.	-
	I.d.	-	2243.6	0.85
	I.d.	-	4664.0	2.00
*R. philippinarum*	I.d.	-	I.d.	-
	I.d.	-	4844.0	1.99
	1237.5	0.75	5296.3	2.05
	**T28**			
	**CNTs**		**CNTs + Cr**	
	**Size (nm)**	**PDI**	**Size (nm)**	**PDI**
*R. decussatus*	I.d.	-	2222.0	1.24
	I.d.	-	I.d.	-
	4343.1	1.76	I.d.	-
*R. philippinarum*	808.4	0.45	7200.7	2.84
	I.d.	-	1096.5	0.43
	954.7	1.42	2209.0	0.85

**Table 2:** Chromium (Cr) concentrations (mg/L) in water collected every week after spiking for each species in control (CTL) and spiked conditions. LOQ is the limit of quantification.

	Cr (mg/L)	
	*R. decussatus*	*R. philippinarum*
CTL	<LOQ	<LOQ
Spiked	3.21 ± 0.37	3.15 ± 0.53

**Table 3:** Chromium (Cr) concentrations in clams (μg/g dry weight (DW)) collected at the end of the experimental period (28 days) and in each tested treatment. Significant differences (*p* < 0.05) among exposure treatments were presented with different letters (uppercase letters for *R. decussatus* and lowercase letters for *R. philippinarum*) and between species with asterisks (*). Values correspond to 3 clam samples *per* aquarium ± standard deviation.

	Cr (μg/g DW)	
	*R. decussatus*	*R. philippinarum*
CTL	1.36 ± 0.58 ^A^	1.17 ± 0.36 ^a^
CNTs	1.26 ± 0.19 ^A^	0.98 ± 0.06 ^a^
Cr	115.55 ± 15.80 ^B*^	48.13 ± 20.85 ^b*^
CNTs + Cr	118.51 ± 9.77 ^B*^	71.6 ± 17.12 ^b*^

## Data Availability

Not applicable.

## Supplementary Materials

The following supporting information can be downloaded at: https://www.mdpi.com/article/10.3390/nano13040690/s1, Methodologies: Biomarkers responses.
