# Supplementary material for: Effects of Carbon Nanoparticles and Chromium Combined Exposure in Native (Ruditapes decussatus) and Invasive (Ruditapes philippinarum) Clams
Source: Nanomaterials (Basel). 2023 Feb 10;13(4):690. doi: 10.3390/nano13040690 (PMC9963187; doi:10.3390/nano13040690)
Supplement: Supplementary file 1 [file nanomaterials-13-00690-s001.zip › nanomaterials-2111705-supplementary.pdf]

S1. Determination of biomarker responses

S1.1. Metabolic activity and energy reserves

Electron transport system activity (ETS) was measured following De Coen and Janssen (1997) and King and Packard (1975) methods. The absorbance was measured at 490 nm in 25 s intervals for 10 min and the activity was obtained using the extinction coefficient ε = 15.9/(mmol/L)/cm. The results were expressed in nmol per min per g of fresh FW.

Biuret method was used to determine protein (PROT) content (Robinson and Hogden, 1940), using bovine serum albumin (BSA) as standard (0–40 mg/mL). Absorbance was measured at 540nm and the results were expressed in mg per g of FW.

S1.2. Indicators of cellular damage

Lipid peroxidation (LPO) levels were measured according to the method described by Ohkawa et al. (1979) through the quantification of malondialdehyde (MDA). Absorbance was read at 535 nm (ε = 156/(mmol/L)/cm). LPO levels were expressed in nmol of MDA per g FW.

S1.3. Antioxidant defenses and biotransformation mechanisms

Superoxide dismutase (SOD) activity was determined according to the method of Beauchamp and Fridovich (1971). For the calibration curve, were used SOD standards (0.25–60 U/mL). After 20 min of incubation at room temperature, the absorbance was measured at 560 nm. The activity was expressed in U per g FW, where U of enzyme activity corresponds to quantity of the enzyme that catalyzes the conversion of 1 μmol of substrate per min.

Catalase (CAT) activity was determined according Johansson and Borg (1988) with modifications performed by Carregosa et al. (2014). The calibration curve was made using standard solutions of formaldehyde (0-150 mM). The reading was performed at 540 nm and the results were expressed as U per g of FW where U indicates the formation of 1 nmol of formaldehyde per min.

Glutathione peroxidase (GPx) was quantified following the method of Paglia and Valentine (1967). The absorbance was read at 340 nm (ε = 6.22/(mmol/L)/cm) in 10 s intervals during 5 min. The results were expressed in U per g of FW where U indicates 1 μmol of NADPH oxidized per min.

Glutathione-S-transferase (GSTs) activity was determined using the extinction coefficient 9.6/(mmol/L)/cm for CDNB and the absorbance was read at 340 nm (Habig et al., 1974). Results were expressed in U per g of FW where U is defined as the amount of enzyme that produces 1 μmol of dinitrophenyl thioether per min.

S1.4. Neurotoxicity

Acetylcholinesterase (AChE) activity was determined according to Ellman et al. (1961) modified by Mennillo et al. (2017) using acetylthiocholine iodide (ATChI, 5 mmol/L) as substrate. The enzyme activity was recorded continuously for 5 min at 412 nm and expressed nmol per min per g FW, using ε = 13.6/(mmol/L)/cm.
